# The Radiotherapy Role in the Multidisciplinary Management of Locally Advanced Vulvar Cancer: A Multidisciplinary VulCan Team Review

**DOI:** 10.3390/cancers13225747

**Published:** 2021-11-17

**Authors:** Luca Tagliaferri, Valentina Lancellotta, Calogero Casà, Simona Maria Fragomeni, Martina Ferioli, Stefano Gentileschi, Anna Amelia Caretto, Giacomo Corrado, Benedetta Gui, Giuseppe Ferdinando Colloca, Maria Antonietta Gambacorta, Alessio Giuseppe Morganti, Giorgia Garganese, Gabriella Macchia

**Affiliations:** 1 Dipartimento di Diagnostica per Immagini, Radioterapia Oncologica ed Ematologia—Fondazione Policlinico Universitario A. Gemelli IRCCS, 00168 Rome, Italy; luca.tagliaferri@policlinicogemelli.it (L.T.); valentina.lancellotta@policlinicogemelli.it (V.L.); benedetta.gui@policlinicogemelli.it (B.G.); giuseppeferdinando.colloca@policlinicogemelli.it (G.F.C.); mariaantonietta.gambacorta@policlinicogemelli.it (M.A.G.); 2 Dipartimento Scienze della Salute della Donna, del Bambino e di Sanità Pubblica, Fondazione Policlinico Universitario A. Gemelli IRCCS, 00168 Rome, Italy; simona.fragomeni@policlinicogemelli.it (S.M.F.); stefano.gentileschi@policlinicogemelli.it (S.G.); giacomo.corrado@policlinicogemelli.it (G.C.); 3 Radiation Oncology Center, IRCCS Azienda Ospedaliero Universitaria di Bologna, DIMES, Alma Mater Studiorum—Bologna University, 40138 Bologna, Italy; m.ferioli88@gmail.com (M.F.); amorganti60@gmail.com (A.G.M.); 4 Università Cattolica del Sacro Cuore, 00168 Rome, Italy; carettoaa@virgilio.it; 5 Gynecology and Breast Care Center, Mater Olbia Hospital, 07026 Olbia, Italy; ggarganese@gmail.com; 6 Dipartimento Scienze della Vita e Sanità Pubblica, Università Cattolica del Sacro Cuore, 00168 Rome, Italy; 7 Unità Operativa di Radioterapia, Ospedale Gemelli Molise, Università Cattolica del Sacro Cuore, 86100 Campobasso, Italy; gabriella.macchia@unicatt.it

**Keywords:** vulvar cancer, radiotherapy, review

## Abstract

**Simple Summary:**

Locally advanced vulvar cancer (LAVC) requires a multidisciplinary management. The aim of this paper is to conduct a review of all relevant studies regarding the role of radiotherapy in LAVC. Based on the available evidence, radiotherapy, with or without concurrent chemotherapy, has a relevant role in neoadjuvant, adjuvant or exclusive treatments. A multidisciplinary and multidimensional assessment can also be useful to identify the most suitable approach, in view of a better treatment personalization.

**Abstract:**

Locally advanced vulvar cancer (LAVC) is a challenging disease, requiring multidisciplinary management. The aim of this review is to propose an integrated clinical approach including radiotherapy (RT) in the multidisciplinary management of LAVC to customize the treatment. A review of the literature was conducted on PubMed, Scopus, and Cochrane library to acquire all relevant studies on RT in LAVC. Based on the available evidence, RT, with or without concurrent chemotherapy, has a relevant role as adjuvant and exclusive treatment or in the neoadjuvant setting. However, multicentric prospective trials are needed to define the best treatment options based on tumor and patient characteristics. A multidisciplinary and multidimensional assessment can also be useful to identify the most suitable approach, considering patients’ age and comorbidities, in view of a better treatment personalization.

## 1. Introduction

Vulvar cancer (VC) is an uncommon disease, usually affecting elderly women and representing about 5% of gynecological cancers [1,2,3].

VC patients require a multidisciplinary evaluation for primary surgery and inguinal lymph node assessment, and potentially adjuvant radiation and/or chemotherapy [4,5,6,7,8]. Radiotherapy (RT) and/or chemotherapy may be considered for primary treatment instead of surgery in cases that would otherwise require radical surgery such as abdominal-perineal resection or exenterative procedures.

As there is no standard treatment algorithm that fits all patients, multimodal clinical approaches, especially in VC involving perineal tissues, have been proposed [8,9,10].

Groin and pelvic adjuvant RT should be administered for any nodal metastasis and/or close/involved margins unfit for further surgery. Large tumors, or those at high risk of surgical morbidity, are candidates for primary (chemo) radiation treatment, and multidisciplinary teams play an important role in the evaluation phase [11,12,13,14,15].

In recent decades, RT has benefited from major technological improvements, with image-guided and intensity-modulated treatments. The use of computed tomography (CT) and magnetic resonance imaging (MRI) for treatment planning allows for the precise delineation of the tumor and surrounding organs (OARs), improving target dose coverage. Modern techniques allow the delivery of comparatively higher doses to the tumor, while minimizing doses to healthy tissue. In several cancers, these improvements lead to a better outcome, in terms of both local control and toxicity rates [16,17,18].

The aim of this review is to summarise the main evidence published in the last 30 years on the role of RT in the multidisciplinary management of locally advanced VC.

## 2. Materials and Methods

### 2.1. Data Sources and Searches

A systematic search was carried out using PubMed (https://pubmed.ncbi.nlm.nih.gov/, first accessed on 15 June 2021), Scopus (https://www.scopus.com/, first accessed on 15 June 2021), and Cochrane library (https://www.cochranelibrary.com/, first accessed on 15 June 2021) to identify full papers reporting on RT in patients with locally advanced VC. We selected the studies including the terms “vulvar neoplasms” and “radiotherapy” as medical subject headings (MeSH) and/or keywords. The Medline search strategy was: [“Radiotherapy” (MeSH) OR “Radiation therapy” (All fields)] AND [“Vulvar Neoplasms” (MeSH) OR “Vulvar Cancer” (All fields)]. To avoid missing relevant studies, we chose this strategy, which is burdened by a high sensitivity and low specificity. All full-text papers published in English and reporting on patients with locally advanced VC treated with RT were identified and reviewed.

### 2.2. Study Selection

The articles that met the following inclusion criteria were retained in the final analysis: (a) clinical prospective or retrospective studies on patients with histological confirmation of primary VC; (b) sample size ≥ 10 patients; (c) RT delivered with or without concurrent chemotherapy in adjuvant, neoadjuvant, or definitive setting; (d) studies published in English between 1997 and 2021; (e) studies reporting oncological outcomes and/or toxicity. Planning studies, case reports, surveys, letters, editorials, book chapters, review articles, and conference abstracts were excluded.

### 2.3. Data Extraction

The citation list of all the included articles was screened independently and in duplicate by two authors (MF, CC) at the title and abstract level to identify other potentially relevant studies without any duplication. Eligible citations were retrieved for full-text review. Uncertainties about inclusion in the review were resolved by an expert radiation oncologist involved in the VUL.CAN board (VULvar CANcer tumor board) (VL). For each study, the following data were extracted: first author’s last name, enrollment period, study design, oncological outcomes and toxicity. All relevant papers were analysed and organised according to the RT setting (adjuvant, neoadjuvant, and definitive RT) and discussed by the VUL.CAN board of our institution, including radiation oncologists, gynaecologists, medical oncologists, plastic surgeons, radiologists, and onco-geriatricians. The preliminary results were finally discussed and validated by two radiation oncologists (LT and MAG) of the first author’s institution and by two radiation oncologists (AGM and GM) and one gynaecologist (GG) from other institutions. The primary outcome was clinical/pathological complete response. Secondary outcomes included local control (LC), disease-free survival (DFS), overall survival (OS), clinical/pathological response and rate of adverse events. A descriptive statistical analysis was conducted of the data available from the published papers.

## 3. Results

A total of 19 studies were included in the analysis and are reported according to their different therapeutic setting [8,11,15,19,20,21,22,23,24,25,26,27,28,29,30,31,32,33,34]. One out all analyzed studies had a randomized prospective design with different treatment strategies in terms of external beam RT (EBRT) techniques and chemotherapy schedules. According to the International Federation of Gynecology and Obstetrics, the majority of cases were stage III and IV (Table 1, Table 2 and Table 3). The FIGO classifications used were: FIGO 1988, FIGO 2009 staging system in one [19] and in six [25,27,29,31,32] papers, respectively. The TNM Classification of Malignant Tumors staging system was used in three studies [11,15,20].

EBRT was delivered by two-dimensional, three-dimensional/intensity-modulated technique and prothon therapy in 148, 2401 and 1 patients, respectively. Median dose to the pelvis/vulva in neoadjuvant, exclusive and adjuvant setting was 47.6 Gy, 66 Gy and 50 Gy, respectively.

Concurrent chemotherapy was based on 5-fluorouracil and cisplatin in three studies [8,19,20], on 5-fluorouracil and mitomycin-c combination in two studies [24,25] and cisplatin alone in three studies [22,26,30].

### 3.1. Neoadjuvant Radio-Chemotherapy

**Figure 1:**
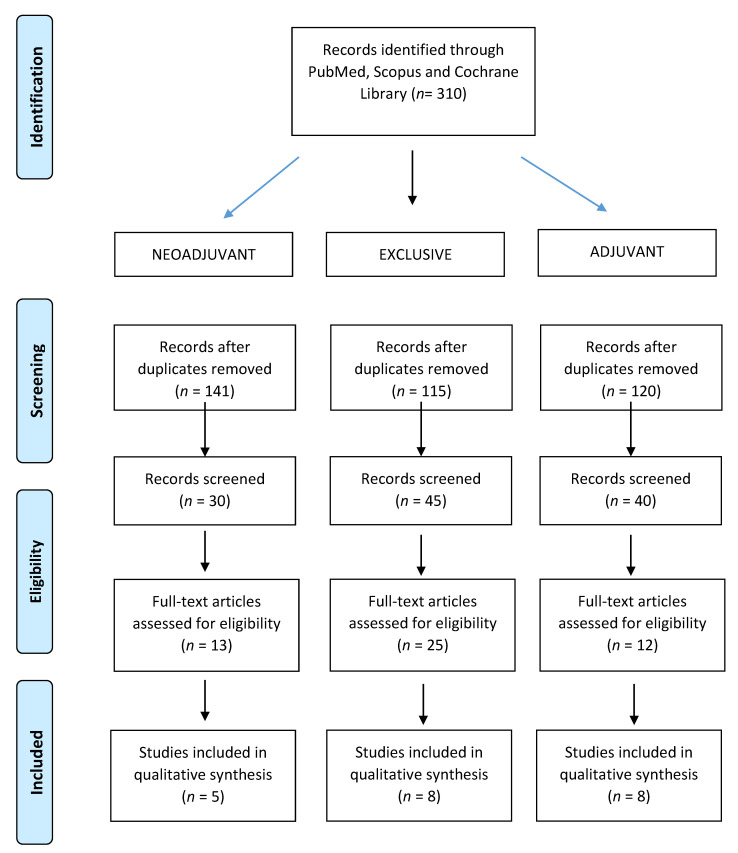
PRISMA Flow-chart for outcomes and toxicity.

From the 310 retrieved papers, five studies reporting data on 782 patients (median age, 68 years; range 88–73.5 years) met our inclusion criteria and included patients who underwent preoperative (chemo) radiation [19,20,21,22,23], as shown in Figure 1.

A complete pathologic response was recorded in 135/229 patients (58.9%). Two-, 3- and 5-year OS was 69%, 57–61% and 57%, respectively [19,20,21,22]. Two- and 3-year DFS was 55% and 65.9%, respectively [19,26].

Acute/late toxicity higher than grade 2 (radiation therapy oncology group (RTOG) or common terminology criteria for adverse events (CTCAE)) was registered in 18 patients as muco-cutaneous vulvar-perineal reaction [20,22,23], diarrhea [22], recto-vaginal fistula [23], hematological toxicity [22,23], chronic perineal pain [23], and hip fracture [23]. There were two treatment-related deaths [22,23].

Postoperative complications were observed in 80/246 (32.5%) operated patients. The majority of cases were infection/necrosis (26 patients), lymphocele (14 patients), chronic lymphedema (14 patients), and the breakdown of vulvar wounds (23 patients), wound evisceration (1 patient), hematoma (1 patient) and femoral artery hemorrhage (1 patient) [19,20,21,22,23].

### 3.2. Exclusive Radio-Chemotherapy

From the 310 retrieved papers, eight studies reporting data on 2722 patients (median age, 70; range 56.5–80 years) met our inclusion criteria [21,22,24,25,26,27,28,29], as shown in Figure 1.

Patients were treated with exclusive radio-chemotherapy for primary carcinoma of the vulva due to severe associated medical problems or extensive local disease that precluded curative surgical resection.

A complete clinical response was recorded in 103/177 patients (58.1%).

Median 5-year OS, LC, DFS rates were 49.9% [24,27,29], 39%, [29] and 45.6% [24,29], respectively. The addition of concurrent chemotherapy to RT resulted in significantly improved relapse-free survival [24], disease-specific survival [24], and OS rates [24,27]. The effect of RCT on OS was evident in both patients with node-positive (*p* < 0.001) and node-negative (*p* < 0.001) disease, as well as in patients age ≤75 years (*p* = 0.008) and age >75 years (*p* = 0.041) [27]. In one study, a significant correlation was recorded between RT dose ≥50 Gy and improved OS [21]. Concurrent radio-chemotherapy also improved OS compared to RT alone when a dose > 55 Gy was delivered [21]. Older age (>60–68 years) was correlated with worse OS [21,27,29], DFS [29], and complete response rate [29]. Conversely, a complete clinical/pathological response was found to be predictive of higher OS rates [22,26].

Acute/late toxicity higher than grade 2 (RTOG or CTCAE) was reported in 68 patients as skin desquamation (27 patients) [22,25,26,29], gastrointestinal/genitourinary toxicity (22 patients) [22,25,29], hematological toxicity (9 patients) [25], vaginal stricture (3 patients) [22], urinary fistula (2 patients) [26], radiation ulcer (4 patients) [28] and femoral radio necrosis (1 patients) [28]. There was only one (0.03%) treatment-related death [27].

### 3.3. Adjuvant Radiotherapy Plus/Minus Chemotherapy

From 310 retrieved papers, eight studies reporting data on 5439 patients (median age, 69; range 65–74.4 years) met our inclusion criteria [8,11,15,30,31,32,33,34], as shown in Figure 1.

Close margin was generally defined as a distance of less than 8 mm between cut tissue edge and invasive tumor.

Recommendations for adjuvant radiotherapy were margins status (close or positive), tumor depth of invasion >5 mm; nodal status (single positive lymph node if metastasis diameter is <2 mm). Recommendations for adjuvant radio-chemotherapy were positive margin, presence of a single positive lymph node if metastases diameter is >2 mm; presence of two or more positive lymph nodes; presence of node with extracapsular extension (ECE).

Five-year OS and DFS were 63% [15,34] and 61.2% [11,34], respectively. Ten-year LC was 41.1% [30]. Statistically significant differences in terms of OS and DFS were recorded between stages (I-II vs. III and IV; *p* < 0.0001) and based on the nodal status (N0 vs. N+; *p* < 0.0001) [35]. Adjuvant RT significantly improved LC- and cancer-specific survival in patients with positive nodes [15,33]. In a multivariable analysis of node-positive patients comparing subjects treated with (*n* = 183) and without adjuvant RT (*n* = 165), and adjusted for age, ECOG, UICC stage, grade, invasion depth, and number of positive nodes, the effect of adjuvant therapy on PFS and OS remained consistent (PFS: HR = 0.58 (95% CI = 0.43 to 0.78, *p* < 0.001), OS: HR = 0.63 (95% CI = 0.43 to 0.91, *p* = 0.01)) [33]. Age ≤ 76 years and RT total dose >54 Gy were significantly associated with better DFS (*p* = 0.044 and *p* = 0.012, respectively) [30,34] and OS (*p* = 0.015 and *p* = 0.015, respectively) [30,32,34]. Five-year overall survival (OS) was highest among patients with one positive node who received radio-chemotherapy (68.1%), compared to 55.9% for adjuvant EBRT and 46.1% for no adjuvant treatment. Survival was likewise highest among the patients with two or more positive nodes who received RCT (49.1%), compared to 29.4% for adjuvant EBRT and 21.2% for no adjuvant treatment [15].

Acute/late > grade 2 (RTOG or CTCAE) toxicity was reported in 25 patients (17.8%) in terms of skin desquamation, diarrhea and leg oedema [8,31].

### 3.4. Interventional Radiotherapy (Brachytherapy)

From the 141 retrieved papers, nine studies reporting data on 177 patients (median age, 67; range 27–93 years) met our inclusion criteria [35,36,37,38,39,40,41,42]. All patients were treated with interstitial IRT. The latter was delivered as a boost after EBRT or as exclusive treatment in primary or recurrent vulvar cancer therapy. Studies evaluating interventional RT (IRT) in the treatment of locally advanced primary VC showed 43.5 median 5-year LC% (range 19–68%), 44.5 median 5-year DFS% (range 44–81%), and 50.5% median 5-year OS rate (range 27–85%) [38,39,40,41,42]. In the treatment of recurrent VC, IRT resulted in 47% 5-year LC [39], 64% (range 56–72%) median 5-year DFS, and 45% (range 33–57%) median 5-year OS [35,36,39].

## 4. Discussion

This review provides, in our opinion, consistent evidence of RT’s efficacy in terms of clinical outcomes across different clinical settings, with acceptable toxicity rates.

The 30% of VC patients presenting with locally advanced disease (T3/T4) may represent a problem regarding the treatment. Ultraradical surgery alone (radical vulvar operation combined with a partial or total pelvic exenterative-type procedure) is associated with a 4.3% mortality rate and 46% disease-free survival [43]. However, although this is not well reported in the literature, there is significant physical and psychological morbidity resulting from these procedures due to a permanent colostomy, urostomy or both [43,44]. In comparison with radical surgery, chemotherapy has been shown to be associated with poor survival and significant treatment-related toxicity [45,46]. RT combined with chemotherapy, followed by organ-sparing surgery has shown efficacy in preventing stoma formation, but is also associated with significant wound-healing problems and treatment-related mortality [24,26,27]. High-quality evidence on neoadjuvant RT was difficult to collect for several reasons, such as a small sample size, the heterogeneity of studies, and the use of different radio-chemotherapy schedules, RT dose fractionation techniques and target definitions. There was no evidence of a survival advantage or reduction in toxicity when neoadjuvant radio-chemotherapy was compared to primary surgery for women with locally advanced VC [47]. In patients with large tumours that can only be treated with anterior and/or posterior exenteration, the complications of neo-adjuvant therapy might outweigh the complications of exenterative surgery. Neoadjuvant therapy is not justified in patients with tumours that can be adequately treated with radical vulvectomy and bilateral groin node dissection alone [48].

Exclusive radio-chemotherapy of locally advanced VC was tested by several studies, based on the results reported in patients treated with neoadjuvant radio-chemotherapy. Compared with an upfront radical surgical approach, definitive radio-chemotherapy allows for organ preservation with good clinical outcomes. Landrum et al. demonstrated that at a median follow-up of 31 months (range from 3–161 months), there were no significant differences in OS and DFS according to treatment group (radio-chemotherapy versus surgery) (*p* = 0.83, *p* = 0.81, respectively) [30]. There was no evidence of a survival advantage when primary surgery was compared to radio-chemotherapy for women with locally advanced VC [48]. There were no studies showing a statistically significant difference in treatment-related adverse effects with the above-mentioned methods of treatment. Due to the sparse data and the relatively high risk of bias in the literature data, no definite conclusions can be drawn [48].

The results of the present study suggest that dose escalation, utilizing modern RT techniques with concurrent chemotherapy in the definitive setting, may lead to an improved response and similar or improved tolerance. In the present systematic review, the median delivered total dose was 61 Gy, as compared to 57.6 Gy in GOG 205 [49] and 47.6 Gy in GOG 101 [23]. The rates of cCR were 48% in GOG 101, 64% in GOG 205, and 58.1% (range 27.8–88%) in the present study. A comparison between these response rates and historic controls from GOG 101 and GOG 205 suggests that a dose–response relationship may exist [25]. The importance of the clinical/pathological response was underlined in one study, showing it to be significantly correlated with improved OS [32]. The high rate of CR achieved by exclusive radio-chemotherapy and the low rate of relapse suggest that the combination of RT and chemotherapy could be synergistic [15]. Furthermore, the combination of RT plus concurrent chemotherapy resulted in significantly improved relapse-free survival [20], disease-specific survival [20] and OS [20,27] compared to RT alone.

Age was a significant predictor of OS [25,26,30,33,36]. When survival in patients younger than the median age of 64 was compared to those aged 65 and older, significant improvements were noted in OS [25,26,30,33,36] as well as PFS [30]. Furthermore, age above 60–68 years also had a detrimental effect on complete response rate [26].

Adjuvant RT is indicated in patients with high risk factors, such as close/involved margins or inguinal lymph node involvement, to decrease the rate of recurrence, and thus improve OS, as shown in our results. Statistically significant improvements in OS and DFS according to low-stage and negative nodal status [31] were found, together with an improved LC in patients with close/involved surgical margins after adjuvant RT [33]. Even in the adjuvant setting, we recorded high clinical outcome rates [32,33,34]. These results, particularly in terms of LC, favorably compare with surgery alone. In fact, even in patients with early VC, surgical resection is associated with local recurrence rates of up to 40%. [50]. Moreover, after surgery that affect tissues worsening tolerance, severe acute/late toxicity was reported in about one third of patients, mainly in terms of skin side effects.

The treatment with IRT that allows for the delivery of a high radiation dose to the tumor, while sparing the surrounding, at-risk organs, with a very sharp dose fall-off, deserves special attention. It is well known that IRT is an effective treatment option for primary and recurrent VC, especially in patients with severe comorbidities and contraindications for surgery [1]. Our analysis confirmed previous findings, which reported encouraging 5-year clinical outcomes, especially considering the preferential selection of most frail patients for IRT [23,45,46,48,51]. Despite these positive data, IRT is rarely considered among the therapeutic options for locally advanced VC patients. It is likely that the rarity of this tumor, lack of widespread experience and expertise, and complexities in performing this treatment technique limit the use of IRT in the majority of RT centers [52,53]. IRT could theoretically also be used as a boost after concurrent radio-chemotherapy to improve LC rates, especially in larger VCs. However, the role and real efficacy of IRT-based boost is largely unproven, with no prospective or randomized controlled trials available in this setting [54].

The small sample size and retrospective design of most studies, along with the wide enrollment interval (1991–2021) and consequent inclusion of patients treated with obsolete RT techniques, represent the limits of our analysis. These issues resulted in a high risk of bias and poor generalizability of the results. However, given the complex target shape, it is challenging to deliver high doses and achieve a uniform target irradiation with the optimal sparing of healthy tissues with this technique. In centers equipped with intensity-modulated treatments, both IMRT and volumetric-modulated arc therapy are increasingly used. However, only a few studies have tested IMRT in patients with locally advanced VC [19,26,55]. For instance, Beriwal et al. reported a reduced irradiation in small-bowel, bladder, rectum, and femoral heads, with 71.0% and 42.8% complete clinical and pathological response rates, respectively and good local control [19]. Delivering a total dose of 70 Gy, Rao et al. achieved a cCR of 61% and a pCR of 44%. Another recognized limitation is the lack of human papilloma virus (HPV) status in the analyzed papers. Several reports investigating the relationship between HPV infection and vulvar carcinoma prognosis reported conflicting results. Nevertheless, the evidence provided by these studies is mainly indirect, as most of them did not provide data on HPV detection [56,57]. Therefore, the design of large databases that can assist in defining patients’ and tumors’ characteristics and allow for greater personalization of treatment strategies appears useful. In this framework, two national data collection projects (OLDLADY-1 and OLDLADY-2) are currently underway in our centers to generate two large databases on exclusive and adjuvant radio-chemotherapy in patients with VC, respectively [58,59]. Despite the difficulties in deriving conclusions as to which clinical or pathological factors are predictors of recurrence and survival in the dose-escalated modern RT technique era, the data on CR and low toxicity are promising.

## 5. Conclusions

Most of the evidence on RT of VCs is low-level, based on retrospective studies. However, in different treatment settings, RT results are quite homogeneously encouraging even if there is clearly room for further improvements, in terms of both treatment outcomes and late sequelae and patient selection. These improvements may derive from prospective and possibly randomized studies, even if the rarity of VCs severely limits their feasibility. In parallel, or alternatively, the design of large databases could allow for the development of predictive models, which are particularly useful for defining individualized treatments based on tumors and patients’ characteristics. To date, the multidisciplinary management of these patients, based on tumor board discussion, represents the broader and more fruitful cooperation possible when choosing the best treatment for each patient.

## Figures and Tables

**Table 1:** Neoadjuvant setting.

Author	Period	Study	Tumor Stage (No of Patients)	Median Age, Years	DFS	OS	Toxicity (G > 2)	Median FU, Months
Beriwal [19]	2002–2011	Mo.	III: 23; IVa: 3	73.5 (37–89)	3-y:65.9%	3-y: 61.2%	2.38%	15 (3–111)
Gaudineau [20]	2001–2010	Mo.	III–IV: 22	74 (51–81)	-	5.1 y	22.7%	2.3 years
Natesan [21]	2004–2012	Mo.	II–III–IV: 639	68	-	3-y: 57.1%	n.a.	21.9
Richman [22]	2012–2019	Mo.	II–III–IV: 24	68 (59–72)	2-y: 55%	2-y: 69%	70.8%	20 (7–36)
Moore [23]	2005–2009	Mu.	III–Iva: 71	-	-	-	74.6%	24.8–

Abbreviations: DFS: disease-free survival; G: grade; Mo: monocentric; Mu: multicentric; na: not available; OS: overall survival; y: year.

**Table 2:** Exclusive setting.

Author	Period	Study	Tumor Stage (No of Patients)	Median Age, Years	DFS	LRC	OS	Toxicity (G > 2)	Median FU, Months	Main Results
Han [24]	1973–1998	Mo.	RT-CT: III: 8; IVa: 2; Rec: 4 RT alone: II: 1; III: 8; IVa: 3	78.5 (35–89)	RT-CT: 5y: 62% RT alone: 5y: 14% *p* = 0.001	-	RT-CT: 5y: 54% RT alone: 5y: 10% *p* = 0.004	N.A.	26 (3.5–273)	RCT resulted in significantly improved relapse-free survival (*p* = 0.01), disease-specific (*p* = 0.03) and overall survival (*p* = 0.04) cCR: 10 patients (71.4%), cPR: 4 patients (28.6%)
Sakanaka [25]	2011–2014	Mo.	II/III/IV:10	62 (58−69)	3-y:80%	3-y:88.9%	3-y:100%	50%	46 (40–54)	cCR: 10 patients (100%)
Rishi [26]	2012–2018	Mo.	III–IV: 26	60.5(38–92)	1-y: 86%	1-y: 85.4%	1-y: 91% 2-y: 62%	34.6%	17.8 (3–53)	CR at 3 months after RT was a predictor for OS (1 yr OS 73% vs. 27%, hazard ratio (HR) 7.1 (95% confidence interval (CI) 1.2–44); *p* = 0.01) Tumor doses > 66 Gy (*p* = 0.03) and prior pelvic radiotherapy (*p* = 0.002) reached significance for development of high-grade soft-tissue toxicity
Natesan [21]	2004–2012	Mo.	II–III–IV: 1047	70	-	-	3-y: 41.7%	N.A.	21.9	The 3-year OS for women treated with primary RT/RCT was 41.7% compared with 57.1% for women treated with RT/RCT + S (*p* < 0.001). Older age (*p* < 0.001), higher Charlson–Deyo score (*p* < 0.001), and AJCC stage N3 versus N0 (*p* < 0.001) were associated with compromised OS Exclusive RCT with doses less than or equal to 55 Gy had a 3-year OS of 33.0% compared with 50.3% for those who received doses of more than 55 Gy (*p* < 0.001).
Rao [27]	2003–2014	Mo.	RT-CT: II: 202; III: 453; IVa:279RT alone: II:99; III: 113; IVa: 76	63 (23–90) 80 (30–90)	-	-	RT-CT: 5-y: 49.9% RT alone: 5-y: 27.4% *p* < 0.001	N.A.	27.1 (6–132)	Age (*p* < 0.001), Charlson–Deyo score (*p* = 0.003), FIGO stage (*p* = 0.030), and chemotherapy (*p* < 0.001) were associated with OS RCT was associated with decreased risk of death compared to RT alone (*p* < 0.001). The effect of RCT on OS was significant in FIGO II (*p* < 0.001), FIGO III (*p* < 0.001), and FIGO IVA (*p* < 0.001), in node-positive (*p* < 0.001) and node-negative (*p* < 0.001) disease, as well as in patients age ≤ 75 (*p* = 0.008) and age >75 (*p* = 0.041) Addition of chemotherapy was associated with a 22% reduction in mortality (*p* = 0.023).
Richman [22]	2012–2019	Mo.	II–III–IV: 25	68 (59–72)	2-y: 81%	-	2-y: 63%	35%	20 (7–36)	cCR at first FU (*p* < 0.01), pCR at the primary site (*p* < 0.01), pCR in inguinal nodes (*p* = 0.04), and pCR in both primary and nodes (*p* = 0.01) predicted for OS Age > 68 year (*p* = 0.01) was detrimental for OS cCR: 22 patients (88%)
Tans [28]	1997–2007	Mo.	IVa: 20	68 (43–87)	1-y: 71% 4-y: 71%	1-y: 75% 4-y: 75%	1-y: 88% 4-y: 65%	45%	42 (6–144)	cCR: 20 patients (72%), cPR: 4 patients (14%)
Alanyali [29]	2000–2011	Mo.	III–IV: 11	68 (28–86)	5-y: 29.2%	5-y: 39%	5-y: 36.4%	25%	-	Older age, poor tumor differentiation, positive surgical margin, and lymphovascular space invasion were found to be important prognostic factors for disease-related outcomes

Abbreviation. CT: chemotherapy; RCT: radio-chemotherapy; cCR: clininical complete response; cPR: clininical partial response; DFS: disease free survival; FU: follow-up; Gy: gray; LC: local control; Mo.: monocentric; N: nodes; NA: not available; Na: not available; pCR: patological complete response; OS: overall survival; y: years; RT: radiotherapy; Rec: recurrence.

**Table 3:** Adjuvant setting.

Author	Period	Study	Tumor Stage (No of Patients)	Median Age, Years	DFS	LRC	OS	Toxicity (G > 2)	Median FU, Months	Main Results
Logar [30]	1997–2004	Mo.	Stage II: 4 Stage III: 33 Stage IVa: 11 n.a.: 3	74.4	10-y: 34.5%	10-y: 41.1%	10-y: 22.7%	n.a.	22.5 (2–203)	Factors that contribute to lower outcome in stage I and II were higher age (mean age 79.9 ± 6.5, *p* = 0.04). ECE had a negative impact on LC (*p* = 0.02). If N+, LC decreased by 60% (*p* = 0.03), and OS as well as DFS decreased by 50% (*p* = 0.2). There was a trend to a better LC with doses > 54.0 Gy (*p* = 0.05).
Kunos [31]	2009	Mo. Randomized	Adjuvant RT III–IV: 59 No Adjuvant RT III–IV: 55	70 (23–89)	n.a.	Adjuvant RT 6y: 59% No Adjuvant RT III–IV: 48%	Adjuvant RT 6y: 36% No Adjuvant RT III–IV: 13%		74	At 6 years, the cumulative incidence of cancer-related death was 29% for RT compared with 51% for pelvic node resection (hazard ratio 0.49, 95% CI 0.28–0.87, *p* = 0.015) Significant univariable association between greater than 20% positive ipsilateral groin nodes and the number of contralateral lymph node metastases (*p* = 0.02), pelvic node metastasis (*p* = 0.06), recurrence (*p* = 0.03), cancer-related deaths (*p* = 0.02), and all-cause deaths (*p* = 0.01)
Tagliaferri [8]	2013–2017	Mo.	Stage II: 2 Stage III: 24	70 (18–87)	2-y: 82% 3-y: 72.4%	2-y: 88.6% 3-y: 79.3%	2-y: 91% 3-y: 91%	14.2%	32 (6–72)	Loco-regional and systemic disease control are favorable, not only in node-negative patients, but also in node-positive patients
Gill [32]	1998–2011	Mu.	Stage III–IV 1797	69 (21–90)	n.a.	n.a.	CT 3-y: 46.9% No CT 3-y: 53.9%		28.3 (11.6–70.6)	Older patients (age > 75 years: HR 4.32, 95% CI 2.94–6.33, *p* < 0.001), patients with greater Charlson–Deyo comorbidity scores (≥2: HR 1.58, 95% CI 1.06-2.35, *p* = 0.026), and higher lymph node involvement (≥4 lymph nodes involved: HR 2.84, 95% CI 2.20–3.67, *p* < 0.001) had a greater risk of death Delivery of adjuvant CT resulted in a 38% reduction in the risk of death (HR 0.62, 95% CI 0.48–0.79, *p* < 0.001)
Mahner [33]	1998–2008	Mu.	Adjuvant RT III–IV: 244 No Adjuvant RT III–IV: 169	67 (30–87)	Adjuvant RT 3-y: 39.6% No Adjuvant RT 3-y: 39.6% *p* = 0.004	n.a.	Adjuvant RT 3-y: 57.1% No Adjuvant RT 3-y: 51.4% *p* = 0.17	n.a.	39.4 (11.8–71.4)	DFS and OS reduction in pts with increasing numbers of N+ (*p* < 0.001). 3-year DFS in N+ receiving adjuvant RT was statistically significantly better compared with N+ patients without adjuvant RT (39.6% vs. 25.9%, *p* = 0.004). 3-year OS rate was statistically not significant (57.7% vs. 51.4%, *p* = 0.17). Adjuvant RT was a statistically significant predictor for cancer-related DFS and OS (adjuvant RT vs. none DFS: *p* = 0.001; OS: *p* = 0.04)
Laliscia [34]	1999–2016	Mo.	IB–II: 17 III–IV: 34	71 (38–86)	5-y: 52%	n.a.	5-y: 63%	n.a.	31 (3–204)	Age < 76 year and RT total dose >54 Gy were significantly associated with better DFS (*p* = 0.0444 and 0.012, respectively) and OS (*p* = 0.015 and 0.015, respectively)
Rydzewski [15]	2004–2014	Mu.	Adjuvant RT III–IV: 974 Adjuvant RCT III–IV: 744 No Adjuvant RT III–IV: 1061	n.a.	n.a.	n.a.	Adjuvant RT 5-y: 29.4–55.9% Adjuvant RCT 5-y: 49.1–68.1% No Adjuvant RT 5-y: 21.2–46.1% *p* < 0.001	n.a.	n.a.	More nodes examined, higher T stage, older age, and more co-morbidities were also associated with worse OS. OS was highest for the RCT group for both patients with one N+ and those with two or more N+. Significantly decreased mortality for patients with 1 N+ who received EBRT (*p* = 0.001), patients with 2 or more N+ receiving EBRT (*p* < 0.001), patients with 1 N+ receiving RCT (*p* = 0.004), and patients with 2 or more N+ receiving RCT (*p* < 0.001).
Parthasarathy [11]	1998–2001	Mu.	Adjuvant RT III–IV: 102 No Adjuvant RT III–IV: 106	65 (29–87) 71 (31–100)	Adjuvant RT 5-y: 77% No Adjuvant RT 5-y: 61.2% *p* = 0.02	n.a.	n.a.	n.a.	n.a.	RT improved the OS of those patients who had a less extensive lymphadenectomy (≤12 lymph nodes removed) from 55.1% to 76.6% (*p* = 0.035). Younger age (*p* = 0.008) is a significant independent prognostic factor after controlling for factors such as year of diagnosis, percent positive nodes, grade of disease, and use of adjuvant RT.

Abbreviation. RCT: radio-chemotherapy; DFS: disease free survival; EBRT: external beam radiotherapy; ECE: extracapsular extension; G: grade; LC: local control; Mo: monocentric; mu: multicentric; N+: positive nodes; na: not available; RT: radiotherapy; OS: overall survival; y: years; FU: follow-up.
